# The Pathophysiology of Gilles de la Tourette Syndrome: Changes in Saccade Performance by Low-Dose L-Dopa and Dopamine Receptor Blockers

**DOI:** 10.3390/brainsci13121634

**Published:** 2023-11-25

**Authors:** Yasuo Terao, Yoshiko Nomura, Hideki Fukuda, Okihide Hikosaka, Kazue Kimura, Shun-ichi Matsuda, Akihiro Yugeta, Francesco Fisicaro, Kyoko Hoshino, Yoshikazu Ugawa

**Affiliations:** 1 Department of Medical Physiology, Kyorin University, Tokyo 181-8611, Japan; 2 Department of Neurology, University of Tokyo, Tokyo 113-8655, Japan; 3 Yoshiko Nomura Neurological Clinic for Children, Tokyo 113-0034, Japan; 4 Segawa Memorial Neurological Clinic for Children, Tokyo 101-0062, Japankimura@segawa-clinic.jp (K.K.);; 5 Section of Neuronal Networks, Laboratory of Sensorimotor Research, National Eye Institute, Bethesda, MD 20892-2510, USA; 6 Department of Human Neurophysiology, Fukushima Medical University, Fukushima 960-1295, Japan

**Keywords:** saccade, basal ganglia, tic, inhibition, blocker, obsessive compulsive disorders

## Abstract

Aim: To elucidate the pathophysiology of Gilles de la Tourette syndrome (GTS), which is associated with prior use of dopamine receptor antagonists (blockers) and treatment by L-Dopa, through saccade performance. Method: In 226 male GTS patients (5–14 years), we followed vocal and motor tics and obsessive–compulsive disorder (OCD) after discontinuing blockers at the first visit starting with low-dose L-Dopa. We recorded visual- (VGS) and memory-guided saccades (MGS) in 110 patients and 26 normal participants. Results: At the first visit, prior blocker users exhibited more severe vocal tics and OCD, but not motor tics, which persisted during follow-up. Patients treated with L-Dopa showed greater improvement of motor tics, but not vocal tics and OCD. Patients with and without blocker use showed similarly impaired MGS performance, while patients with blocker use showed more prominently impaired inhibitory control of saccades, associated with vocal tics and OCD. Discussion: Impaired MGS performance suggested a mild hypodopaminergic state causing reduced direct pathway activity in the (oculo-)motor loops of the basal ganglia–thalamocortical circuit. Blocker use may aggravate vocal tics and OCD due to disinhibition within the associative and limbic loops. The findings provide a rationale for discouraging blocker use and using low-dose L-Dopa in GTS.

## 1. Introduction

The basal ganglia (BG) and the BG–thalamocortical circuit play important roles in the purposeful initiation and inhibition of voluntary movements, including eye movements. Although the pathomechanism of Gilles de la Tourette syndrome (GTS) remains to be clarified, dysfunction in these structures is considered to underlie its pathophysiology. One of the prevailing current views states that involuntary movements, such as tics, are caused by the emergence of aberrant foci or focal hyperactivity within the basal ganglia–thalamocortical circuit, which disinhibits the downstream structures involved in the generation of movements, such as the thalamus and the cerebral cortex [1]. Recent views associate the emergence of aberrant focus with the phasic dopamine release within the striatum [2]; the effectiveness of dopamine receptor antagonists (dopamine blockers) for controlling tics is consistent with this view, although dopamine agonists may provoke tics.

Several parallel loops of the BG–thalamocortical circuit are implicated in the generation of various clinical manifestations of GTS, such as the motor circuit including the motor cortex for simple tics, the associative circuit including the dorsolateral prefrontal cortex for complex tics, and the limbic circuit including the limbic and paralimbic cortices for obsessive–compulsive disorders (OCDs) [3]. The involvement of multiple loops is also supported by the fact that deep brain stimulation of the parafascicular nucleus of the thalamus alleviates the clinical symptoms of GTS, which is in line with known anatomical connections.

Motor tics involve the face–eye region most frequently [4]. Since the BG–thalamocortical loop responsible for oculomotor control lies between the motor and associative circuits, GTS patients would be expected to manifest abnormal control of eye movements. Indeed, GTS patients can present with blepharospasm, sustained gaze deviation [5], and involuntary eye movements presumably representing eye tics [6]. Interestingly, the volume of caudate, implicated in the generation of voluntary saccades, has been reported to be inversely correlated with tic severity [7].

Recent reports suggest that dopamine agonists and partial dopamine agonists (aripiprazole) are useful in some cases of GTS for suppressing tics [8,9], clinically lending support to the hypothesis of an up-regulated D2 receptor, but stronger evidence would be provided by treatment with L-Dopa. Meanwhile, neuroimaging studies have provided controversial evidence for receptor supersensitivity or up-regulation in GTS [10,11,12]. While aripiprazole with its partial agonist D2 receptor can stabilize the neural transmission according to the dopaminergic neuronal tone of the individual patients, it also has a variety of side effects, sometimes severe, including difficulty in sleeping (insomnia), sleepiness, anxiety and restlessness, headache, nausea or vomiting, indigestion/constipation, and blurred vision. In contrast, the greatest benefit of a low dose of L-Dopa is its safety without major side effects, and secondly, the well-established mechanism of action in treating post-synaptic dopamine receptor supersensitivity (for a review, see [13]). The impact of antipsychotic medication on functional brain systems may be primarily through activation in the frontal and parietal eye fields, showing improved function in attentional and sensorimotor systems, as well as the cerebellum. This aspect of pathophysiology could be addressed using oculomotor paradigms, especially saccades, in the patients, subserved by the basal ganglia and the cerebellum that receive command from these cortical areas.

In GTS, oculomotor findings, such as impaired voluntary initiation and inhibition of saccades, have also been observed, ranging from abnormalities of both voluntary and reflexive saccades [14], delayed latencies of sequences of memory-guided saccades (MGS) and antisaccades, reduced peak velocity of the antisaccades with normal directional error in the antisaccade task [15], and normal latency but shorter duration of saccades in visually guided saccades (VGS) [16].

The first systematic investigation addressing the pathophysiology of eye movements in GTS was performed by LeVasseur et al. [17] using the anti- and prosaccade tasks with or without a delay period interposed between the appearance of the target and the go signal. The latency of saccades was prolonged in both tasks, suggesting impaired voluntary initiation of saccades. Moreover, whereas the directional error rate in the delayed antisaccade task (a voluntary saccade task in which the participants have to make a saccade away from the presented target) was within the normal range, the timing error in the delayed task increased (i.e., saccades were initiated during the delay period). On this basis, they suggested that while the inhibitory control of saccades toward novel stimuli was normal in GTS, the excitability of the superior colliculus (SC) gradually became abnormally enhanced during the delay period, making it harder for GTS patients to withhold the execution of planned motor programs. This gradual increase in SC excitability may be produced by an aberrant focus in the basal ganglia disinhibiting the downstream structures, presumably due to a phasic dopamine release [10].

In contrast, some saccade studies have arrived at a different, albeit not mutually exclusive, view on the pathophysiology of GTS. Although they also reported impaired suppression of saccades, Nomura et al. [6] found prolonged latency, hypometria, and a reduced MGS success rate in GTS patients, similar to early-stage Parkinson’s disease and Segawa disease [18], suggesting a primary hypodopaminergic state. Segawa [19] and Nomura et al. [6] explained the emergence of tics by the activation of post-synaptic dopamine D2 receptors showing hypersensitivity in the presence of a decreased basal dopamine level in the basal ganglia. Furthermore, analysis of phasic activity during rapid eye movement sleep in GTS [20] suggested compensatory up-regulation of dopamine receptors in response to the decreased activity of dopamine at the terminals of the nigrostriatal dopamine neuron [4,19]. Wolf et al. [21] studied five pairs of adult monozygotic twins discordant for Tourette syndrome severity who showed increased iodobenzamide binding to D2 receptors in the caudate nucleus, suggesting D2 receptor supersensitivity that correlated with the clinical severity of the patients.

The impairments in voluntary initiation and suppression of saccades are thought to reflect dysfunctions of the inhibitory–disinhibitory mechanisms of the BG. The processing for MGS, a voluntary saccade, mainly takes place in the frontal lobe, from which the motor signal is emitted directly or via the caudate nucleus to the SC (the direct pathway of the BG circuit); a phasic reduction from the high resting firing rates of the substantia nigra pars reticulata temporarily releases the saccade cells in the recipient SC, resulting in the generation of voluntary saccades (see [22] for a review)). For VGS, the parietal eye field, including the posterior parietal cortex, mainly integrates visuospatial information to generate a motor signal that is sent to the SC via the parietal lobe–SC pathway [23]. SC serves as the common terminal for these two types of saccades and an organizing center for determining the magnitude and direction of saccades, with converging commands arriving through the basal ganglia–SC pathway and cortex–SC pathways [22,24,25]. In addition, we also studied the inhibitory control of saccades, another important function of the BG, by means of the frequency of saccades to cues that are presented in MGS.

The present study aimed to clarify the pathophysiology underlying GTS through saccade performance, which can be used to probe the dysfunction of BG disorders. Using VGS and MGS tasks, we recorded the saccade performance of 110 GTS patients and compared it with 26 age-matched normal participants, taking into account the large age-related changes in saccade parameters occurring, especially between 5 and 14 years of age.

Clinically, by looking at the effects of dopamine receptor blockers and L-Dopa on the clinical symptoms of the patients at the initial visit to the Clinic and follow-up, we tested the hypothesis of whether the use of dopamine blockers would aggravate rather than improve some of the clinical symptoms in GTS as expected if a basal hypodopaminergic state underlies GTS. Conversely, L-Dopa or dopamine agonists would improve this state and would, consequently, decrease tics. A preliminary account of this paper has appeared in abstract form [26].

## 2. Methods

### 2.1. Participants

**Figure 1:**
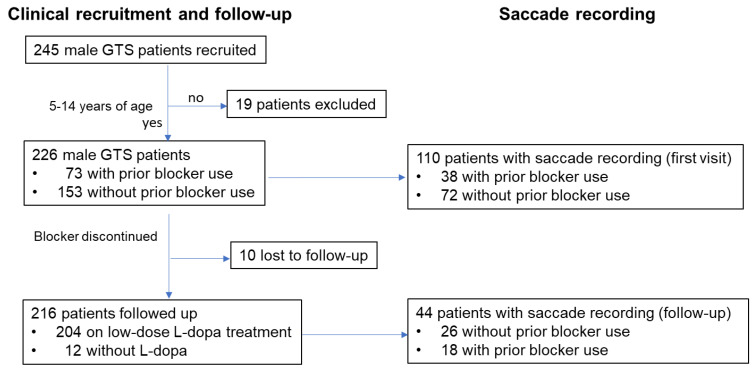
Study flow diagram.

For this investigation, we initially studied 245 male GTS patients, recruited at the first visit to Segawa Neurological Clinic for Children (hereafter, “the Clinic”) in the period from October 1998 to October 2008 (patient age: 5–42 years, Figure 1).

The analysis of this study was unfortunately truncated midway due to the death of one of the leading doctors (Dr. Masaya Segawa) participating in this study. The impact was so tremendous that it was only some time after his death that we could resume the analysis after the research team in the Clinic was reorganized.

The inclusion criteria of patients in this study were GTS patients whose age was 5 or more and who showed informed consent to participate. The exclusion criteria were those with hearing loss that interfered with verbal communication, hand motor symptoms not allowing the subjects to press the button used for the oculomotor task (as described later), and cognitive/perceptive impairment that prevented the subjects from understanding the task procedure, or severe involuntary movements including tics that kept them from being seated for over an hour and/or performing the task properly. GTS was diagnosed according to previously proposed criteria (DSM-IV-TR criteria for the diagnosis of Tourette Syndrome, American Psychiatric Association). Among them, 73 had used dopamine receptor antagonists (haloperidol, a dopamine D2 antagonist, in all cases; hereafter termed “blockers”) and 172 had never used blockers at the first visit to the Clinic. In this study, we restricted analyses to patients between 5 and 14 years of age (9.8 ± 3.8 years) because there were relatively few patients outside this range. We thus focused on 226 male GTS patients; among them, 73 had used dopamine receptor antagonists (blocker users), while 153 were drug-free prior to visiting the Clinic (for clinical information on participants, see Table 1).

Of the blocker users, some had also used other drugs, including antiepileptic, anticholinergic, and antipsychotic drugs (valproate 2, carbamazepine 2, clonidine 2, biperidene 1, paroxetine 1). The patients without prior blocker use had not used any other drugs prior to visiting the Clinic, except for one patient who was taking carbamazepine. The exclusion of these patients did not essentially change the results reported below. Saccade performance (see below) was recorded as part of a clinical assessment. Informed consent was obtained in the form of an opt-out on the website, according to the procedures approved by the ethics committee of Segawa Neurological Clinic for Children (approval number SMNCC21-09). Those who rejected were excluded.

### 2.2. Clinical Assessment of Participants and Follow-Up

As will be shown in the results since, based on our clinical observations, we noted that blockers work adversely on the clinical symptoms of GTS patients, as well as on their saccade performance, patients were stopped at the first visit to the Clinic if they had been used (blocker users). Of the 226 GTS patients, 10 were lost to follow-up. A total of 204 among the remaining 216 patients were started on low-dose levodopa without dopa-decarboxylase inhibitors (L-Dopa) at a dose of 0.5 mg/kg, while 12 GTS patients were not. Consequently, the consequence clinical symptoms of the remaining 216 GTS patients were followed up in the Clinic for a period of 8.0 years on average from the time of the first visit. The two patient groups were comparable in age at onset, age at the first visit, age at follow-up, and follow-up duration (Table 2).

Since standard scales, such as the Yale Global Tic Severity Scale and the Yale–Brown Obsessive Compulsive Scale, could not be implemented in many of the patients due to time constraints, we classified the severity of vocal and motor tics as well as the OCD of the patients on a scale of 0 to 3 based on their influence on daily activities (for vocal and motor tics: 0: no tics, 1: mild tics not interfering with activities of daily living (ADL), 2: moderate tics that interfere but are compatible with ADL and/or job, 3: severe tics that largely restrict ADL; for OCD: 0: no OCD, 1: mild OCD not interfering with ADL, 2: moderate OCD that interferes but is compatible with ADL and/or job, 3: severe OCD that largely restricts ADL; here, motor tics imply all types of tics, such as nose wrinkling, head twitching, eye blinking, lip biting, facial grimacing, shoulder shrugging, other tics of the limbs). These clinical subscores were used for assessing the changes in severity of symptoms during follow-up (see below).

### 2.3. Saccade Recording

Among the 226 patients, saccade recordings were made in 145 GTS patients, as well as 42 normal participants 6 to 24 years of age. Of the 145 patients, statistical analyses were restricted to the age range of 8 to 14 years; that is, 110 patients (38 patients with prior blocker use at least once in the Clinic and 72 drug-naïve patients). Since the number of participants with saccade recording was relatively small outside this age range, younger participants were often unable to perform the task properly, and the three participant groups were age-matched (patients with blockers: 10.9 ± 1.8 years, patients without blockers: 10.6 ± 1.5 years, normal participants: 11.4 ± 1.8 years).

Among these participants, in 44 GTS patients (with prior blocker use: 18 patients, age 12.3 ± 2.5 years at first test; without prior blocker use: 26 patients, age 12.5 ± 2.0 years at first test; no difference in age at first test: *p* = 0.6983), saccade recordings were made twice during the follow-up period: once at the first visit and the second time at a later period separated by 6.0 ± 2.5, and 6.1 ± 2.7 years for the blocker use and non-use groups, respectively (no difference in follow-up duration: *p* = 0.9246). Although, considering the long interval between consecutive saccade recordings, the change in saccade performance may be affected by development or brain maturation in the patients; the follow-up period was almost comparable for the two groups of subjects.

Although we also studied female patients, the number of patients was considered too small for further analyses, and the results were excluded. Although their data are not shown, essentially similar findings as those for male participants were obtained.

**Figure 2:**
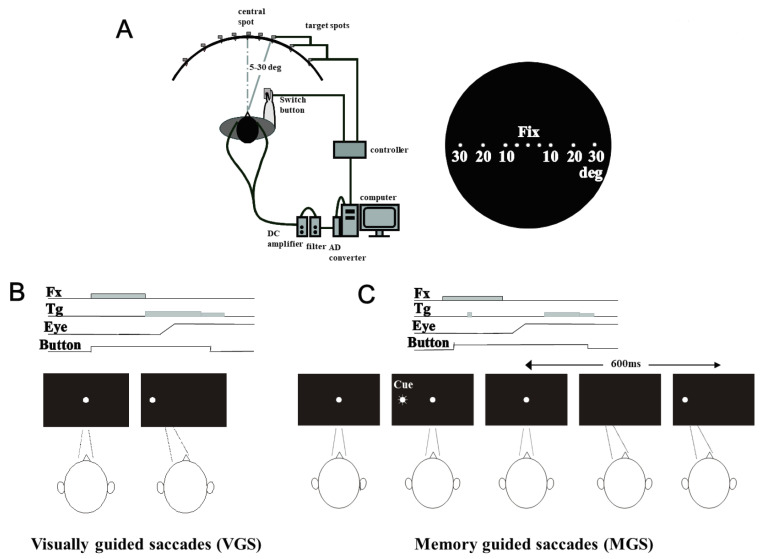
Experimental setup: (**A**) the electrode and adhesive tape used for recording EOG (**B**), electrode placement (**C**), and oculomotor tasks used in the present study (left bottom: VGS, right bottom: MGS). Fx: fixation point, Tg: target, Eye: eye position, Button: button press.

The experimental setup has been described previously ([18]; Figure 2A).

Briefly, DC electro-oculography (EOG) was recorded with 5 Ag-AgCl gel electrodes (2 horizontal outer canthi for recording horizontal eye movements, 2 vertical above and below the right eye for recording vertical eye movements, and 1 ground on the forehead; vertical electrodes were mainly used for monitoring eye blinks). EOG gain was adjusted to the target point at 20 deg left or right. The signals were fed to a DC amplifier (AN-601G, Nihon-Kohden, Japan), low-pass filtered at 20 Hz, and then digitized (500 Hz). The patients were instructed to stop all medications at least 12 h before recording since they are known to influence saccade parameters [27].

Eye movement calibration was performed before each test session. EOG gain was adjusted to a target point at 20 deg left or right; while the participants fixated on this spot, we adjusted the EOG gain so that the eye position displayed on the computer monitor matched the target position displayed on the screen. The gain of EOG was continuously monitored throughout the experiment, and recalibration was performed for adjustment when necessary throughout the experiments. Thus, when calibrated, EOG data are known to be roughly linear over a range of 5 to 30°, and the resolution of our data was 0.5°. Our method has been shown to achieve a good correlation with recordings obtained via a video-based eye-tracking system (Eyelink II, SR Research Ltd., Kanata, ON, Canada) and used in a number of published studies [18].

### 2.4. Behavioral Paradigms

We used VGS and MGS tasks. In VGS (Figure 2B), the fixation point was turned on, and the participants had to fixate on it. It was turned off after a period of 1500 to 2000 ms, and simultaneously the target point was turned on randomly 5, 10, 20, or 30 deg to the left or right. We instructed the participants to foveate the target as quickly as possible.

In MGS (Figure 2C), while the participant fixated on the central spot, a peripheral stimulus (“cue”) appeared for a brief period of 50 ms. The participants maintained fixation until the spot was turned off (delay period, 1.6–2.4 s), at which time they made a saccade to the spatial location where the cue had appeared. The target spot turned on again 600 ms after the offset of the fixation point. Saccades unintentionally made to the cue during the delay period were called *saccades to cue*.

## 3. Data Analysis

Saccade parameters (latencies and accuracies of VGS and MGS, MGS success rate, frequency of saccades to cue) were determined offline. Four parameters were determined offline for each saccade: onset latency, amplitude, duration, and peak velocity. The onset of an eye movement was defined as the time when velocity and acceleration exceeded predetermined values (28°/s and 90°/s^2^, respectively). Eye movement was accepted as a saccade based on its velocity and duration. After the onset, the velocity had to exceed 88 °/s, and this suprathreshold velocity had to be maintained for at least 10 ms. The end of an eye movement was considered to have occurred when the velocity decreased below 40 °/s. The total duration had to exceed 30 ms. Records contaminated by noise and those with onset latency of <60 ms were excluded from the analysis.

The latency of VGS was measured from the time of target presentation, whereas MGS was measured from the time of extinction of the central fixation spot. We also counted the number of successful MGS trials in which saccades were made within the time limit of 600 ms and, therefore, were not VGS. For each participant, the proportion of such successful trials among the total MGS trials was termed the *MGS success rate* as an index of voluntary initiation of saccades. We also calculated the proportion (frequency) of trials with the frequency of saccades to the cue among MGS trials, reflecting the inhibitory control of saccades. The accuracy of the first saccade amplitude was expressed as the ratio (percentage) to the target eccentricity. Before the statistical assessment, all parameters (VGS latency and accuracy, MGS latency and accuracy, frequency of saccades to cue) were collapsed across eccentricities since the effect of eccentricity was not the main interest of this study.

## 4. Data Analysis and Statistical Assessment

Statistical analyses were conducted using SPSS software (ver 28.0.1, SPSS Japan, Tokyo). For all analyses, the significance criterion was set at *p* < 0.05. To identify the effect of prior use of blockers on clinical symptoms during follow-up, we compared the clinical subscores between patients with prior use of blockers and those without at the first visit and follow-up (after quitting blockers). Data from 73 patients with prior blocker use and 153 patients without were entered into a one-way analysis of variance (ANOVA), with group as the between-group factor and age as the covariate. Where necessary, we corrected for sphericity by the Greenhouse and Geisser correction. *Post hoc* analysis by Bonferroni’s method was performed to correct for multiple comparisons to determine what differences contributed to the significance detected (to compare among blocker use group before and after follow-up, no blocker group before and after follow-up).

Second, to observe the effect of L-Dopa on clinical subscores during follow-up, data from 204 patients on L-Dopa and twelve patients without were entered into a one-way ANOVA to compare the scores among four groups (Table 2, L-Dopa group before and after follow-up, no L-dopa group before and after follow-up).

Third, to characterize the saccade abnormalities in GTS patients with and without blockers relative to normal participants, we compared the saccade parameters (latencies of VGS and MGS, frequency of saccades to cue, MGS success rate) at the first visit to see whether there were any significant differences among patients who had been on blockers (38 patients) and those without (72 patients) and 26 age-matched normal control participants. The saccade parameters were entered into a repeated measures ANOVA with the group (3 levels, GTS patients with or without blockers, and normal control) as a within-participant factor.

We also compared the changes in saccade parameters during follow-up with reference to blocker use. Saccade data of 44 GTS patients (18 with prior blocker use and 26 patients without) were entered into a repeated measures ANOVA with prior blocker use as a between-participant factor (2 levels, use and non-use of blocker) and time (2 levels, initial visit and follow-up).

Finally, to test the relationship between the scores of GTS symptoms and saccade parameters, we correlated the clinical scale (motor tic, vocal tic, and OCD subscores) with individual saccade parameters at the first visit (latencies and accuracies of VGS and MGS, frequency of saccades to cue, and success rate of MGS) across all participant groups in GTS patients both with and without blockers using the Spearman’s rank correlation. Correlations among the individual saccade parameters were investigated similarly.

Considering the saccade parameters as a function of clinical subscores, the saccade parameters were entered into a two-way ANOVA with factors of a clinical scale (4 levels: 0, 1, 2, 3 each for motor tic, vocal tic, and OCD subscores) and the participant group (2 levels, GTS patients with and without prior blocker use).

## 5. Results

### 5.1. Effect of Prior Blocker Use on Clinical Subscores and Their Changes during Follow-Up

**Figure 3:**
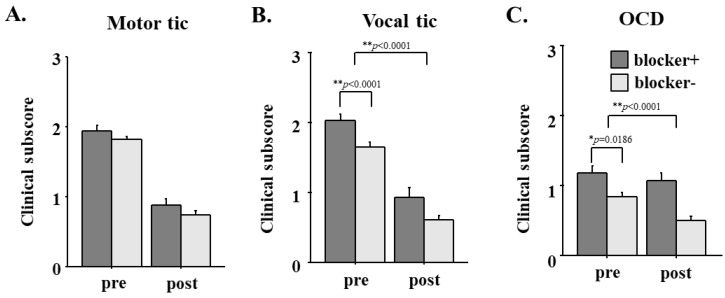
The effect of prior blocker use on clinical subscores at the first visit and follow-up. (**A**) motor tic, (**B**) Vocal tic, (**C**) OCD. Clinical subscores of motor and vocal tics and OCD are compared at the first visit (pre) and at follow-up (post). Black bars indicate patients with prior blocker use and gray bars indicate patients without blocker use. Error bars indicate standard error.

The clinical subscore of motor tics was initially comparable in patients with and without prior blocker use (*p* = 0.0943; Figure 3A, left figure).

The overall subscore of motor tics improved significantly during the follow-up period (effect of time: F = 295.780, *p* < 0.0001; interaction between participant group X time: F = 0.123, *p* = 0.7267). At the end of follow-up for 7.5 years, the motor tic subscore was still comparable between patients with and without prior blocker use (*p* = 0.3792).

In contrast, the clinical subscores of vocal tics and OCD were initially significantly higher in patients with prior blocker use than in patients without prior use (vocal: *p* < 0.0001; OCD: *p* = 0.0186, for vocal tics and OCD, respectively; Figure 3B,C). Both the clinical subscores of vocal tics and OCD decreased significantly during the follow-up period (*p* < 0.0001, *p* = 0.0002), which was similar for patients with and without blockers (effect of time: vocal: F = 285.966, *p* < 0.0001; OCD: F = 14.880, *p* = 0.0002, interaction between participant group X time: vocal: F = 1.258, *p* = 0.2633; OCD: F = 3.547, *p* = 0.0611). At the end of follow-up, the significantly higher vocal tic and OCD subscores in patients with blockers persisted relative to patients without (*p* = 0.0117, *p* = 0117 = 0.0004).

### 5.2. Effect of L-Dopa on Clinical Subscores during Follow-Up

**Figure 4:**
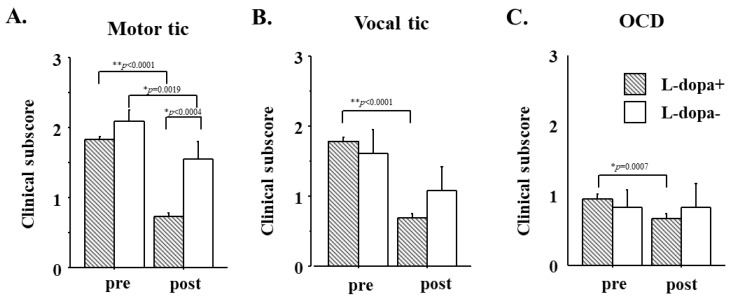
The effect of L-Dopa use on clinical subscores. (**A**) motor tic, (**B**) Vocal tic, (**C**) OCD. Clinical subscores of motor and vocal tics and OCD are compared at the first visit (pre) and at follow-up (post). Bars with slanted stripes indicate patients who were started on L-Dopa at the first visit to the Clinic and white bars indicate patients who were not. Other conventions as in Figure 2.

Patients who were started on low-dose L-Dopa at the first visit to the Clinic and those who were not initially showed comparable clinical subscores of motor tics (Figure 4A), vocal tics (Figure 4B), and OCD (Figure 4C; *p* = 0.4202, *p* = 0.0591, *p* = 0.1457, respectively for motor tics, vocal tics, and OCD; Figure 4).

The clinical subscore of motor tics, vocal tics, and OCD in the L-Dopa group showed a significant improvement (reduction) during follow-up (effect of time: motor: F_1,203_ = 350.072, *p* < 0.0001, vocal: F_1,203_ = 349.011, *p* < 0.0001, OCD: F_1,203_ = 24.022, *p* = 0.0007) for subscores of vocal and motor tics and OCD, respectively), whereas the clinical subscores in the no L-Dopa group showed a significant change only for the motor and vocal subscores. They showed a decreasing trend and a failing significance for the vocal tic and a non-significant change for the OCD subscores (motor: F_1,10_ = 17.500, *p* = 0.0019, vocal: F_1,10_ = 4.865, *p* = 0.0519, OCD: F_1,10_ = 0.129, *p* = 0.7287 for the respective subscores). At the end of the follow-up period, the motor tic was significantly lower in the L-Dopa group than in the no L-Dopa group, whereas the vocal tic showed a trend to be slightly but not significantly lower in the L-Dopa group. The OCD subscore was again comparable between the two groups (*p* < 0.0004, *p* = 0699, *p* = 0.9427, respectively).

### 5.3. Saccade Abnormalities in GTS

**Figure 5:**
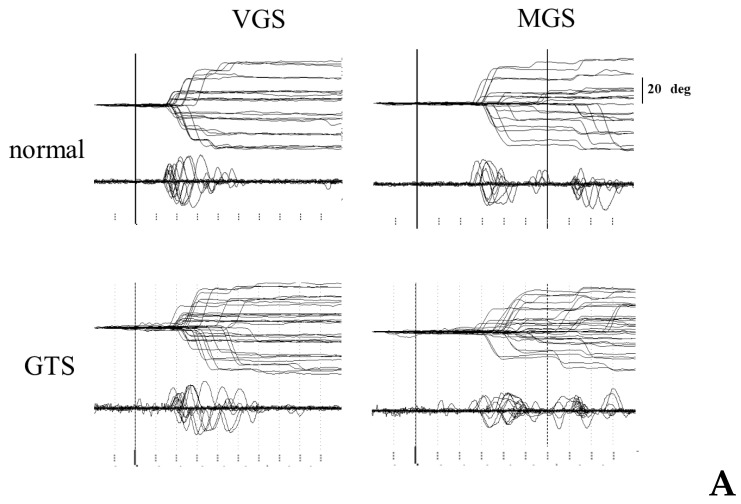

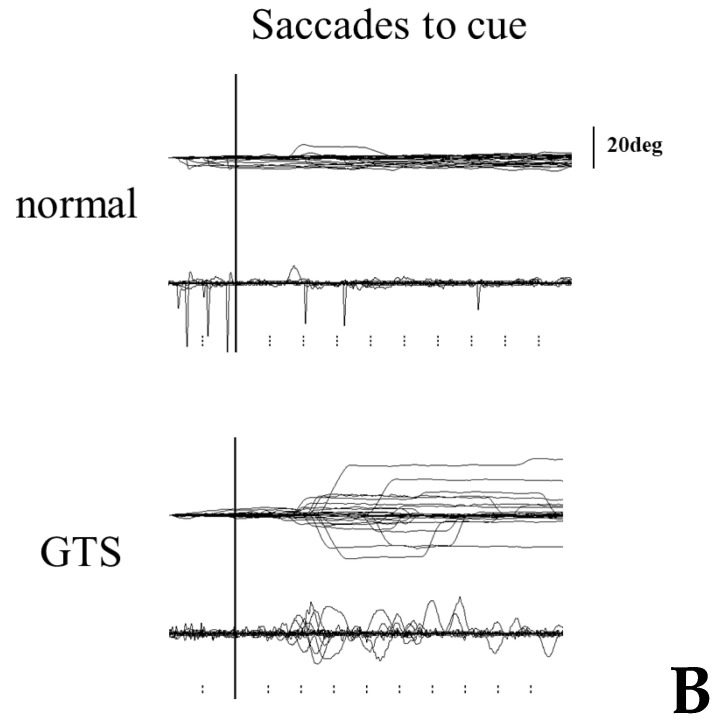
Traces of VGS, MGS (**A**), and saccades to the cue (**B**) A. Traces of VGS and MGS in a normal participant (top row) and a GTS patient. Saccadic eye movements (20–30 trials) are shown as changes in eye position (upper traces) and velocity (lower traces), time-locked to the presentation of the target (VGS) or the offset of the central fixation point (MGS). The abscissa is the time axis and the ordinate gives the angle (or velocity). Traces are time-locked to the offset of the central fixation spot in each task. Tic marks are given at an interval of 100 ms. B. Inadvertent eye movements made during the delay period of MGS (saccades to cues) in a normal participant (top row) and a GTS patient (bottom row). Traces are time-locked to the appearance of the cue in the MGS task.

Figure 5A shows typical examples of MGS and VGS traces recorded in a GTS patient (lower row) and an age-matched normal participant (upper row). In this GTS patient, the latency of VGS in most trials was similar to normal participants, although there were some trials with delayed onset, especially for large target eccentricities, and some trials with small target eccentricities showed short latencies. In contrast, the onset of MGS was overall apparently delayed compared to normal participants. In these patients, the saccade traces showed hypometria compared with normal participants, in which the gaze often reached the target location in two or more steps, which was more prominent for MGS than for VGS.

Patients with GTS sometimes inadvertently made a saccade toward the cue presented in the MGS task, although they were instructed not to (Figure 5B). A saccade toward the cue was more frequently observed than in normal participants.

### 5.4. Comparison of Saccade Parameters in GTS Patients with and without Blocker Use

**Figure 6:**
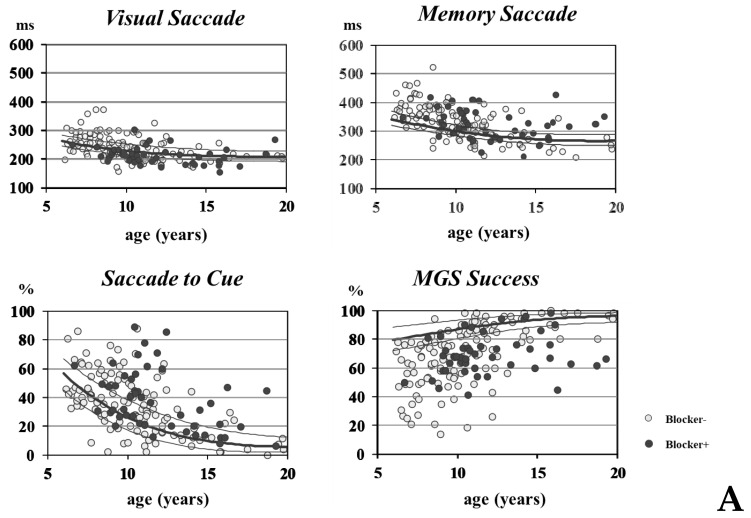

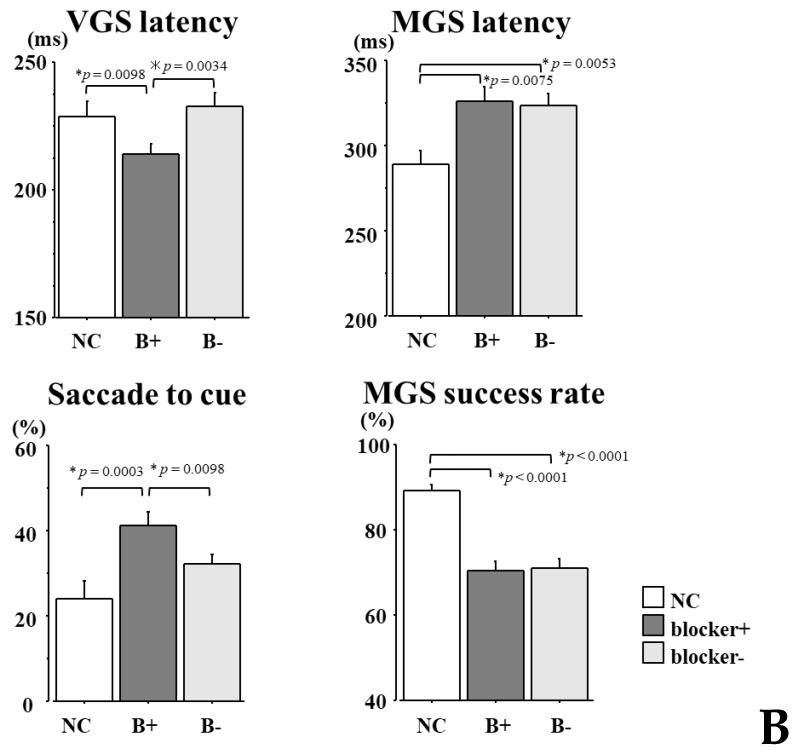
(**A**) Saccade parameters in GTS patients with and without prior blocker use. Saccade parameters are plotted against the age of the patients. The black curves in each figure show the normal range (75th, 50th, and 25th percentiles). The light gray dots represent data of individual patients without prior use of dopamine blockers. Dark gray dots denote patients who had been treated with dopamine blockers. (**B**) The effect of dopamine blocker use on saccade parameters at the first visit. Comparison of saccade parameters at the first visit between normal control (NC) participants and GTS patients with (B+, dark gray bars) and without prior blocker use (B−, light gray bars). Asterisks denote significant differences with corresponding *p*-values. Error bars indicate standard errors.

Saccade abnormalities at the first visit to the Clinic were statistically compared between GTS patients with and without prior blocker use and normal control participants. Figure 6A plots the saccade parameters in individual participants against the age of patients with and without prior use of blockers. The black lines indicate the 75th, 50th, and 25th percentiles of the normal range in the control participants, respectively. All saccade parameters exhibited large age-related changes, especially between 5 and 14 years of age. The distribution of VGS latency (upper left figure) showed a slight shift toward a shorter range, whereas saccades to the cue shifted slightly toward a higher range (lower left figure), especially for patients with prior blocker use relative to the normal range. The distribution of MGS latency shifted toward a longer range (upper right figure), and the MGS success rate shifted toward a lower range (lower right figure) for both patients with or without prior use of blockers relative to normal participants.

The above trend was corroborated by statistical analysis. For VGS latency, there was a significant effect of the participant group (normal participants, patients with and without blockers; Table 3, Figure 6B, upper left figure).

*Post hoc* analysis showed that patients who had been taking blockers showed a significantly shorter VGS latency compared with the control participants and patients without blocker use (patients with blockers vs. normal participants: *p* = 0.0098; patients with blockers vs. without blockers: *p* = 0.0034), whereas VGS latency was comparable between normal participants and patients without blocker use (*p* = 0.7108). Thus, in patients with blockers, VGS latency deviated from normal ranges, even compared with patients who had not been taking blockers.

The frequency of saccades to the cue increased in comparison with normal participants, especially in patients with prior use of blockers. The effect of the participant group on the frequency of saccades to the cue was also significant for the three groups (Figure 6B, lower left figure). Post hoc analysis showed that this was due to the frequency being significantly higher in patients who had been taking blockers relative to normal participants (*p* = 0.0003) and patients without blockers (*p* = 0.0098). The latter two groups were not statistically different (*p* = 0.0766). Therefore, along with VGS latency, patients who had been taking blockers showed an impaired ability to inhibit saccades relative to normal participants and patients without blockers. The accuracy of VGS was lower for patients with blockers compared to normal participants (*p* = 0.0029), whereas it was lower but not significantly after multiple comparisons for patients without blockers compared to normal participants (*p* = 0.0268 after correction for multiple comparisons). Patients with and without prior blocker use were comparable (*p* = 0.1959).

In both patients with and without blockers, the latency of MGS was significantly prolonged and the MGS success rate was significantly reduced compared to normal participants (Figure 6B, upper and lower right figures; an ANOVA showed a significant effect of participant group for both MGS latency and MGS success rate; post hoc analysis showed that both patients who had and had not been taking blockers showed a significantly longer MGS latency compared with control participants (patients with blockers vs. normal control: *p* = 0.0075, patients without blockers vs. normal control: *p* = 0.0053), and a significantly lower MGS success rate (patients with blockers vs. normal, patients without blockers vs. normal control: *p* < 0.0001), whereas the latter two groups were comparable (*p* = 0.8144)). The accuracy of MGS was comparable in the three groups (*p* > 0.3 for all comparisons among groups). Patients with and without prior blocker use were also comparable (*p* = 0.1959).

Thus, saccade parameters indexing the ability of the voluntary initiation of saccades were comparable in GTS patients, regardless of whether they were previously treated or not by blockers, whereas both patient groups differed significantly from normal participants. Parameters indicating the inhibitory control of saccades (i.e., saccades to the cue) were significantly more impaired in GTS patients who had used blockers than those without (see Section 6).

### 5.5. Changes in Saccade Parameters during Follow-Up in Patients with or without Prior Use of Blockers

**Figure 7:**
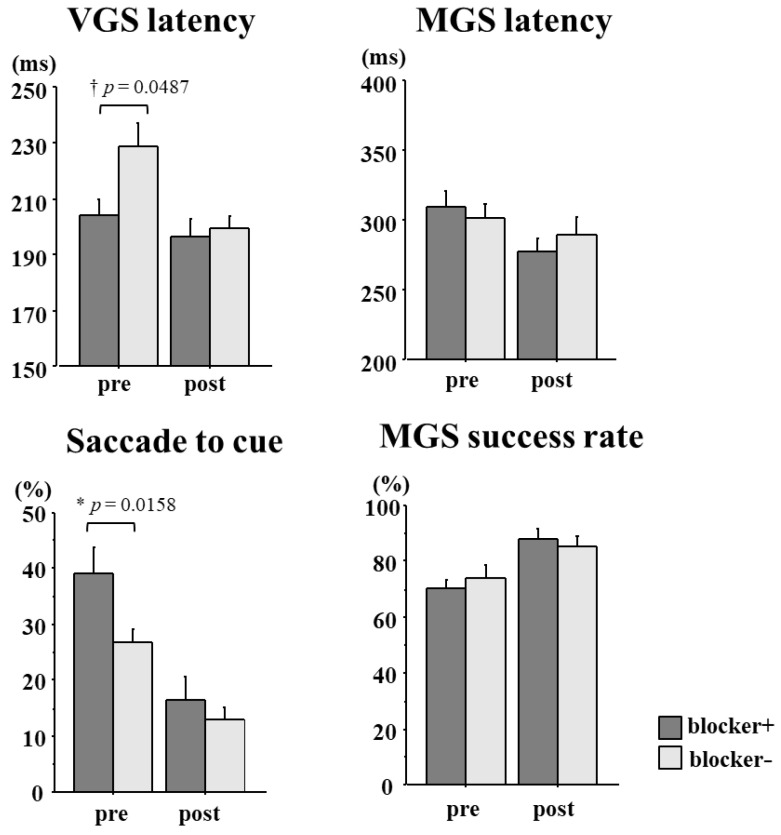
Changes in saccade parameters during follow-up. Saccade parameters were compared at the first visit (pre) and at follow-up (post). Dark gray bars indicate patients with blocker use and light gray bars indicate patients without blocker use. Error bars indicate standard errors. Asterisks indicate significant differences, and daggers indicate a trend for difference.

Saccade parameters were compared between GTS patients with and without prior use of blockers, in whom saccade recordings were made twice during the follow-up period (Figure 7, Table 4).

The frequency of saccades to the cue was initially significantly higher in patients with blocker use (*p* = 0.0158) but decreased for both groups, reaching a similar level at follow-up (*p* = 0.3078). Similarly, VGS latency in GTS patients with blockers was initially shorter relative to GTS patients without blocker use (*p* = 0.0487) but decreased significantly and became comparable for both groups at follow-up (*p* = 0.3078), although it was still somewhat shorter for blocker users. Both measures indicated that the impaired inhibition of voluntary saccades was initially worse for patients with blocker use but became comparable at follow-up.

In contrast, MGS latency and MGS success rate at the first visit were not affected significantly by prior blocker use (Table 4). MGS latency decreased and the MGS success rate increased significantly during follow-up for both GTS patient groups. Thus, measures of the voluntary initiation of saccades remained comparable for both GTS groups at the first visit and follow-up (MGS latency: first visit *p* = 0.5835, last visit *p* = 0.4171; MGS success rate: first visit *p* = 0.5105, last visit *p* = 0.5858). Unfortunately, we were not able to follow up on the saccade parameters in a sufficient number of patients with regard to L-Dopa use; that is, before and after they started low-dose L-Dopa therapy.

### 5.6. Association between GTS Symptoms and Saccade Parameters

We looked at how GTS symptoms are associated with saccade parameters by investigating the correlation between symptom subscores and individual saccade parameters at the first visit across all participant groups, including GTS patients with and without blocker use (Table 5A). The motor subscore showed a trend for negative correlation with MGS latency (r = −0.141, *p* = 0.0661) and VGS accuracy (r = −0.132, *p* = 0.087). There was a strong significant correlation between the vocal tic subscore and the frequency of saccades to the cue (r = 0.197, *p* = 0.0098). The OCD subscore showed a trend for a positive correlation between MGS accuracy (r = −0.131, *p* = 0.0888), frequency of saccades to the cue (r = 0.138, *p* = 0.0734), and MGS success rate (r = −0.15, *p* = 0.0514).

We performed an ANOVA to test whether GTS subscores significantly affected or had an effect on the individual saccade parameters (Table 5B). The motor tic subscore showed a trend for effect on MGS latency that did not reach significance. The vocal tic subscore showed a significant effect on the frequency of saccades to the cue, whereas it showed a trend of effect on the success rate of MGS. The OCD subscore showed a significant effect on the frequency of saccades to the cue, as well as on the success rate of MGS, whereas it showed a trend of effect for the accuracy of MGS. Meanwhile, the OCD subscore was positively associated with the frequency of saccades to the cue and was negatively associated with the MGS success rate. Together with the results of the above correlation analysis, the vocal subscore was most strongly positively associated with the frequency of saccades to the cue. The motor subscore was negatively associated with the MGS latency, but this did not reach significance.

Finally, in GTS patients with and without prior blocker use, we examined the correlation between saccade parameters (Table 5C). VGS latency showed a significantly positive correlation with the frequency of saccade to the cue (r = 0.154, *p* = 0.0449) and a negative correlation with MGS accuracy (r = −0.376, *p* < 0.0001) and MGS success rate (r = −0.455, *p* < 0.0001). MGS latency showed a significant positive correlation with the frequency of saccade to the cue (r = 0.436, *p* < 0.0001), while it showed a significantly negative correlation with MGS accuracy (r = −0.595, *p* < 0.0001) and MGS success rate (r = −0.689, *p* < 0.0001). The MGS success rate was significantly positively correlated with MGS accuracy (r = 0.655, *p* < 0.0001) and negatively correlated with the frequency of saccades to the cue (r = −0.469, *p* < 0.0001). VGS accuracy correlated significantly negatively with the frequency of saccades to the cue (r = −0.162, *p* = 0.0345).

## 6. Discussion

Here, we showed that clinically, patients who had been on dopamine blockers exhibited a more severe degree of vocal tics and OCD but not motor tics than patients who were not on dopamine blockers at the first visit. At follow-up, motor and vocal tic symptoms, as well as OCD, improved in both patient groups. However, the higher subscores for vocal tics and OCD persisted for blocker users relative to drug-naïve patients.

Consistent with our previous report on oculomotor performance in GTS patients [6], the latency of MGS was significantly prolonged, and the success rate of MGS was reduced compared with normal participants in patients who were not using dopamine blockers prior to visiting the Clinic. In contrast, VGS latency tended to be slightly shortened, and the frequency of saccades to the cue increased, although the difference did not reach significance. However, significant differences were found in these saccade parameters when comparing patients who were using blockers prior to visiting the Clinic and those who were not. Importantly, the findings were robust, even when taking into account the age-related changes in oculomotor function during development.

Finally, comparing patients who were prescribed low-dose L-Dopa therapy with those who were not, both groups initially showed comparable scores of clinical symptoms, but the L-dopa group showed lower scores of motor and vocal tics at follow-up.

### 6.1. Mild Dopamine Deficiency Underlies GTS

The direct pathway of the basal ganglia circuit is responsible for the initiation of voluntary saccades, such as MGS (see Introduction). Thus, the prolongation of MGS latency along with the decreased MGS success rate is consistent with mild basal dopamine deficiency in GTS, leading to hypofunction of the direct pathway of the basal ganglia circuit. At first, this finding is somewhat unexpected. Since dopamine receptor blockers are widely used to control tics in GTS, we would have expected a hyperdopaminergic state or an abnormally heightened response to dopamine in the central nervous system.

While MGS latency was prolonged in GTS patients relative to age-matched normal participants, suggesting a dopamine-deficient state, it was not significantly different between patients with and without prior blocker use (Figure 6B). This suggests that prior blocker use was not associated with a more severe dopamine-deficiency state as viewed from saccade performance.

Although several other neurotransmitters are implicated in the pathophysiology of GTS, dopaminergic transmission is still considered to play a major role among them. Early studies have reported a low level of homovanillic acid (HVA), a dopamine metabolite, in the cerebrospinal fluid, which suggests a low basal dopaminergic state in GTS [28]. However, later nuclear imaging [10,11,21,29] and postmortem studies [2,30] are not necessarily concordant in terms of the hypodopaminergic or hyperdopaminergic state regarding the dopamine content, synthesis, or release or in dopamine receptors. Clinically, GTS patients exhibit clumsiness of rapid alternating pronation–supination movements of the arms and induced rigidity in the contralateral arm, which responds to oral L-Dopa, suggesting hypofunction of the nigrostriatal dopaminergic system [4]. Therefore, the oculomotor findings are consistent with a basal hypodopaminergic state that underlies GTS, at least in terms of saccade recordings, even when considering the developmental oculomotor change.

Mild dopamine deficiency with dopamine receptor up-regulation may be reversed by low-dose L-Dopa, as indicated by its effect on motor tics. Actually, the effect of dopamine may well work in a reverse U-shape fashion, and intermediate doses may show a maximal effect. Hence, dopamine partial agonists work effectively and decrease transmission when there is excess dopamine but enhance it when levels are low. However, it is important that the essence is dopamine.

### 6.2. Impaired Inhibitory Control of Saccades in GTS

GTS patients with prior blocker use showed an increased frequency of saccades to the cue compared with normal participants, although the difference in the frequency just failed to reach significance when drug-naïve GTS patients were compared with normal participants (Figure 6B). Since we asked the participants to withhold saccades during the delay period of MGS, the increased frequency of saccades to the cue would imply impaired inhibitory control of saccades. The impaired inhibition of saccades in addition to the prolonged latency of MGS has also been found in patients with early-stage Parkinson’s disease [18] and Segawa disease (hereditary progressive dystonia with marked diurnal fluctuation) [31], in which mild dopamine deficiency is also postulated and was also considered to be explained by mild dopamine deficiency.

Multiple pathways are implicated in the inhibitory control of saccades. These pathways include the direct inhibitory projection of the frontal eye field, altered basal ganglia output from the substantia nigra pars reticulata, omnipause neurons of the midline pontine reticular formation, and fixation neurons in the SC (see [18] for a review). In the absence of pathological changes within the brainstem in GTS and in view of the accumulating literature on the dysfunction of the basal ganglia–thalamocortical pathway in GTS, it is likely that the intrinsic pathology within the inhibitory oculomotor circuits in the brainstem may not be the main reason for impaired saccade inhibition. Rather, the latter may be ascribed to functional impairment of the inhibitory circuit induced by the abnormal input from outside the brainstem, for which altered basal ganglia output through the substantia nigra pars reticulata may play an important role in SC disinhibition. The altered basal ganglia output may derive either from the decreased function of the indirect pathway, the decreased function of the hyperdirect pathway, or the increased function of the direct pathway. We have observed that the function of the direct pathway decreased, which leaves only the two former mechanisms. Thus, the results above were taken to reflect the SC disinhibition in GTS patients with prior blocker use as a result of insufficient output arriving from the basal ganglia. This latter finding has, in fact, been demonstrated experimentally. A reversible blockade of monkey substantia nigra pars reticulata produced irrepressible saccades to the side contralateral to the blockade [24,25].

In contrast, the shortened latency of VGS may also reflect impaired inhibitory control of the saccade. Although VGS may not be directly triggered by visual inputs to SC, the shortened latency of VGS could reflect the enhanced excitability of the SC. For neurons in the caudal SC to fire (so that saccade occurs), they must be released from the inhibition by the basal ganglia. Thus, the shortening of VGS latency may reflect SC disinhibition from basal ganglia inhibition, as postulated for saccades to the cue. However, since VGS latency did not show a significant negative correlation and actually showed a positive correlation with the frequency of saccades to the cue in this study, in contrast to what we found in Parkinson’s disease patients in our previous study [18], the mechanism for shortened VGS latency may differ from that postulated in Parkinson’s disease; namely, functional compensation from neural structures outside the basal ganglia. Alternatively, it is also possible that direct signals sent from the frontal or parietal eye fields (frontal or parietal eye fields) to the SC hyperactivate the oculomotor network in the brainstem, leading to the shortened latency of VGS.

As mentioned in the Introduction, one possible explanation for the increased occurrence of saccades to the cue in GTS may be that a mild upward regulation or hypersensitivity of D2 receptors as a response to dopamine deficiency may plausibly lead to SC disinhibition in GTS [6]. However, neuroimaging studies have been inconsistent regarding the existence of D2 receptor hypersensitivity. Instead, with some variability, recent studies concur on the increases in the number of dopamine receptors, high concentrations of dopamine transporters (DATs), and increased intrasynaptic dopamine release [28]. Singer et al. speculated that an overactive dopamine transporter or central abnormality may lead to an alteration in phasic dopamine release, which, in turn, results in a hyper-responsive spike-dependent dopaminergic system [11]. A reduction in tonic (basal) dopamine, thought to be due to an overactive dopamine transporter system, could result in a system with high concentrations of dopamine receptors and an increased phasic release of dopamine. This could, in turn, lead to the aberrant focus, as proposed by Albin and Mink [1], which would cause tics through the disinhibition of neural structures downstream from the basal ganglia. The same situation could explain the finding that the motor tic subscore at the first visit was negatively associated with the MGS latency (Table 5B), implying that the stronger the motor tic symptoms, the shorter the MGS latency. When aberrant foci are formed within the motor circuit of the basal ganglia, it could lead to an increase in motor tics on the one hand, and the tendency for disinhibition of neural structures downstream from the basal ganglia, such as SC inhibited by substantia nigra pars reticulata, which could lead to shortened MGS latency, on the other hand. Other possible hypotheses for the generation of tics in GTS include the intrinsic abnormalities of the output neurons of the basal ganglia, or alternatively, abnormal synaptic effects impinging on the output neurons.

The vocal tic and OCD subscores were positively associated with the frequency of saccades to the cue (Table 5B), which, in turn, were enhanced in patients with prior blocker use (Figure 5B). Thus, the pathophysiology underlying the increase in saccades to the cue may be related to the increase in vocal tics and OCD. Conversely, the non-use of blockers was associated with lower vocal and OCD subscores, with a lower frequency of saccades to the cue (Figure 7). In contrast, the motor tic subscore was not significantly different in GTS patients, both with and without prior blocker use (Figure 3).

### 6.3. Effect of Dopamine Blockers on Saccade Parameters

Importantly, blocker use was associated with a more prominent impairment of saccade inhibition; that is, the increased frequency of saccades to the cue and the shortening of VGS latency (Figure 6), suggesting that blocker use aggravated the inhibitory control of saccades. The blocking of D2 receptors by dopamine receptor antagonists, reducing the availability of D2/3 dopamine receptors, can lead to an increased occurrence of saccades to the cue by suppressing the indirect pathway, which would reduce the inhibitory influences of basal ganglia on downstream neural structures [12].

Alternatively, it is also possible that direct signals sent from the frontal or parietal eye fields (frontal or parietal eye fields) to the SC hyperactivate the oculomotor network (ON) in the brainstem. This possibility was also suggested in Parkinson’s disease patients, but in this case, there was a significant negative correlation between the frequency of saccades to the cue and VGS latency, but this was not the case in the present study.

As discussed above, the increased frequency of saccades along with the shortened latency of VGS may have resulted because inhibitory output from the basal ganglia is abnormally low. This low output of the basal ganglia may explain not only why prior blocker use aggravates the inhibitory control of saccades but also why other symptoms of GTS patients worsen in blocker users. While oculomotor symptoms may be due to the insufficient inhibitory output of the substantia nigra pars reticulata, non-oculomotor or even affective symptoms (e.g., vocal tics and OCD) may result from the insufficient inhibitory output of the internal segment of the globus pallidus (GPi). This may explain why patients with prior use of blockers presented with a higher degree of vocal tics and OCD, along with the more prominent changes in the saccade parameters indexing saccade inhibition.

In contrast, neither MGS latency nor the success rate of MGS were affected by the prior use of dopamine receptor blockers. The prolongation of MGS latency was equally noted in patients with and without prior blocker use, and thus, blocker use may not directly affect the function of the direct pathway of basal ganglia, which is mainly mediated by the dopamine D1 receptors. Together, these findings may reflect that while dopamine blockers work through the dopamine D2 receptors, they do not affect the basal level of dopamine transmission, as reflected in the function of the direct pathway effected through the dopamine D1 receptor.

### 6.4. Effect of Blocker Use on the Clinical Symptoms of GTS and Their Relevance to GTS Pathophysiology

Patients with prior blocker use were initially presented with a more severe degree of motor and OCD than those who had never used blockers, consistent with the notion of reduced inhibitory influence of the basal ganglia in blocker users (see above). After a follow-up period of 7.5 years after dopamine blockers were discontinued, however, patients with blocker use showed improvement in all these symptoms, although the difference in severity persisted at follow-up, whereas saccade parameters became comparable between the two groups (Figure 2 and Figure 6). Thus, the dopamine blockers worked adversely on clinical symptoms, such as vocal tics and OCD. Although vocal tics and OCD symptoms significantly improved with age, they persisted even after the discontinuation of blockers. Thus, while some adverse effects of dopamine blockers (e.g., vocal tics and OCD) were not only temporary, they can persist long after withdrawal, similar to extrapyramidal symptoms, such as dyskinesia. Part of the improvement may be ascribed to the developmental changes during these ages; reduced D2 receptor function with advancing age might correspond to the spontaneous reduction in tic severity [32]. Considering its long-term influence, blocker use should be strongly discouraged in GTS patients.

### 6.5. Use of Low-Dose L-Dopa Treatment on Clinical Severity

Although the use of L-Dopa has been largely avoided as the treatment of GTS, the present study suggests dopaminergic medication as a rational treatment for GTS based on the physiological evidence of a low dopaminergic state. Indeed, the efficacy of dopaminergic drugs has been indicated in some studies. While an early report described the acute worsening of tics with L-Dopa, other studies did not consistently observe exacerbation or even note the suppression of tics following L-Dopa exposure [33]. Reduced tic severity has also been reported in GTS patients treated with pergolide [34] and ropinirole [35] when given at much lower doses than those prescribed for Parkinson’s disease, although presynaptic rather than post-synaptic mechanisms of action are postulated for this effect. Similarly, the dosage commonly used for Parkinson’s disease would actually overcompensate for the dopamine deficiency. We considered low-dose L-Dopa in GTS to be sufficient for correcting the developmentally up-regulated dopamine receptors in the presence of mild basal dopamine levels and ameliorating the resulting tic symptoms. In the long term, this would also improve the clinical symptoms of GTS patients.

Consistent with this prediction, GTS patients started on L-Dopa showed a more prominent improvement of motor and vocal tics compared to those who were not at follow-up, although both groups initially showed comparable severity scores. These findings attest to the effectiveness of low-dose L-Dopa in normalizing the underlying pathophysiology of GTS and leading to the eventual suppression of tics. However, OCD persisted even after treatment by L-Dopa was introduced; OCD may not be as readily reversible as motor and vocal tics after the withdrawal of blockers or the introduction of L-Dopa, for which the involvement of other neurotransmitters, such as serotonin, may be postulated.

In summary, the findings of the present study clearly showed that dopamine receptor blockers aggravated the symptoms of GTS patients, such as vocal tics and OCD. With development, subscores of motor and vocal tics, as well as OCD, all significantly improved, but the higher vocal tics and OCD subscores in prior blocker users persisted relative to non-users. This suggested that prior use of dopamine receptor blockers may have impaired the function of the oculomotor, as well as the associative circuits, but not the motor circuit, to which the dysfunction of neurotransmitters other than dopamine may also be related. Meanwhile, saccade recordings showed that the pathophysiology of GTS represents a mild basal hypodopaminergic state, improved by L-Dopa administration. L-Dopa use resulted in an improvement of the motor and vocal tic scores, as well as an improvement of the MGS latency and MGS success rate, regardless of blocker use or non-use. This suggests that the use of L-Dopa may have improved the sensorimotor and oculomotor circuits of the basal ganglia–thalamocortical pathway relative to its non-use, but not the affective pathway. In contrast, the OCD subscore was not improved by L-Dopa. The longer VGS latency and the higher saccade to the cue rate in blocker users relative to non-users disappeared with L-Dopa use, but this change may have been due to development rather than L-Dopa use. Indeed, L-Dopa use does not change the frequency of saccades to the cue and increases the latency of VGS in Parkinson’s disease patients [18].

Although one limitation of our study is that the number of participants in the no L-Dopa group was small and that the observations made were largely retrospective, it also provided clinical evidence not only for discouraging the use of blockers but also for using low-dose L-Dopa in treating GTS. Clinical issues related to, for example, the optimal dosage of L-Dopa and the duration of treatment warrant future investigation by studying a larger number of participants using a prospective design.

## 7. Conclusions

Impaired MGS performance in GTS suggested a mild hypodopaminergic state causing reduced direct pathway activity in the (oculo-)motor loops of the basal ganglia–thalamocortical circuit. Blocker use may aggravate vocal tics and OCD due to disinhibition within the associative and limbic loops. The findings provide a rationale for discouraging blocker use and using low-dose L-Dopa in GTS.

## Figures and Tables

**Table 1:** Clinical information of GTS patients.

	GTS with Blocker	GTS without Blocker	F_1,224_
No. of participants	73	153	
Age at onset (years)	5.7 ± 2.1	5.4 ± 2.2	*p* = 0.3303
Age at first visit (years)	11.7 ± 4.1	8.9 ± 3.3	*p* < 0.0001
Age at follow-up (years)	20.4 ± 8.1	16.0 ± 6.1	*p* < 0.0001
Follow-up duration (years)	8.7 ± 6.3	7.1 ± 4.6	*p* = 0.0288
No. of participants with tics	47	94	* *p* = 0.795
No. of participants with sleep disorders	44	41	* *p* < 0.0001
WISC-III	86.1 ± 16.7	94.1 ± 14.6	*p* = 0.2151
No. of patients taking other drugs	9 (valproate 2, carbamazepine 2, clonidine 2, biperidene 1, paroxetine 1, methylphenidate 1	carbamazepine 1	

Asterisks indicate significant differences between GTS patients with and without blocker use.

**Table 2:** Saccade parameters of normal controls and GTS patients with and without prior blocker use.

	GTS with L-Dopa	GTS without L-Dopa	Difference between Groups (F_1,214_)
No. of participants	204	12	
Age at onset (years)	5.4 ± 2.2	5.4 ± 2.4	*p* = 0.9760
Age at the first visit (years)	9.5 ± 3.2	10.5 ± 4.5	*p* = 0.2998
Age at follow-up (years)	16.8 ± 5.8	18.9 ± 12.0	*p* = 0.2648
Follow-up duration (years)	7.4 ± 4.4	8.4 ± 8.8	*p* = 0.4551
No. of participants with tics	28	4	*p* = 0.1826
No. of participants with sleep disorders	73	5	*p* = 0.782

**Table 3:** Statistical results comparing saccade parameters between patients with and without blockers as well as normal participants (ANOVA).

Parameter		Normal	GTS (Blocker +)	GTS (Blocker −)
VGS	Latency (ms)	235.92 ± 28.48	211.68 ± 22.52	232.81 ± 41.31
	Accuracy (%)	94.45 ± 3.7	90.26 ± 6.01	91.67 ± 5.61
MGS	Latency (ms)	289.35 ± 39.76	325.81 ± 50.45	323.57 ± 57.67
	Accuracy (%)	80.23 ± 11.33	81.44 ± 9.72	79.1 ± 13.88
Success rate of MGS (%)		88.94 ± 8.36	70.2 ± 13.55	70.97 ± 19.5
Frequency of saccades to the cue (%)		23.92 ± 22.22	41.97 ± 20.09	31.76 ± 16.98
Reaction time (ms)		288.11 ± 38.47	321.82 ± 48.39	326.98 ± 55.4

**Table 4:** Statistical results for changes in saccade parameters during follow-up (ANOVA) in patients with and without prior blocker use.

	Effect of Time		Effect of Blocker		Time X Blocker	
	F_1,42_	*p*	F_1,42_	*p*	F_1,42_	*p*
VGS latency	14.630	0.0005 *	1.601	0.2141	4.180	0.0485 *
MGS latency	7.155	0.0106 *	0.035	0.8522	1.778	0.1896
MGS success	23.701	<0.0001 *	0.025	0.8751	1.162	0.2872
saccades to cue	41.869	<0.0001 *	4.797	0.0344 *	2.470	0.1239

*: *p* < 0.05.

**Table 5:** Correlation analysis between GTS subscores and saccade parameters (A) and statistical results for the ANOVA in GTS patients.

**A.** Correlation analysis.							
		**Latency**		**Accuracy**		**Frequency of Saccades to the Cue**	**MGS Success Rate**
		**VGS**	**MGS**	**VGS**	**MGS**		
Vocal tic subscore		−0.064	0.057	0.071	0.038	0.197 *	−0.105
Motor tic subscore		−0.08	−0.141 ^†^	−0.132 ^†^	0.023	0.024	0.068
OCD subscore		0.033	0.092	−0.057	−0.131 ^†^	0.138 *	−0.15 *
**B.** ANOVA results.							
**Vocal tic subscore.**							
		**Effect of Subscore**		**Effect of Group**		**Subscore X Group**	
		**F_1,167_**	***p***	**F_1,167_**	***p***	**F_1,167_**	***p***
VGS latency		0.023	0.8809	0.375	0.5413	1.048	0.3075
MGS latency		1.415	0.2358	2.798	0.0962 ^†^	1.82	0.1791
VGS accuracy		1.531	0.2177	1.189	0.2772	0.702	0.4034
MGS accuracy		0.008	0.9296	1.153	0.2844	0.272	0.6024
Frequency of saccades to the cue		8.65	0.0037 *	1.929	0.1667	1.741	0.1888
MGS success rate		3.306	0.0708 ^†^	1.65	0.2007	0.745	0.3892
**Motor tic subscore.**							
		**Effect of Subsore**		**Effect of Group**		**Subscore X Group**	
		**F_1,167_**	***p***	**F_1,167_**	***p***	**F_1,167_**	***p***
VGS latency		0.558	0.4561	1.72	0.1915	0.002	0.9654
MGS latency		2.944	0.088 ^†^	0.002	0.9665	0.058	0.8107
VGS accuracy		1.593	0.2087	0.979	0.3239	0.767	0.3826
MGS accuracy		0.009	0.9245	0.702	0.4034	0.7254	0.124
Frequency of saccades to the cue		0.136	0.7131	0.09	0.7647	0.08	0.7782
MGS success rate		0.413	0.5212	0.086	0.7693	0.0003	0.9865
**OCD subscore.**							
		**Effect of Subscore**		**Effect of Group**		**Subscore X Group**	
		**F_1,167_**	***p***	**F_1,167_**	***p***	**F_1,167_**	***p***
VGS latency		1.523	0.219	6.59	0.0111 *	0.11	0.7404
MGS latency		2.406	0.1228	1.506	0.2214	0.509	0.4767
VGS accuracy		3.611	0.112	0.858	0.3556	0.712	0.4
MGS accuracy		2.986	0.0858 ^†^	0.335	0.5635	0.409	0.5235
Frequency of saccades to the cue		5.067	0.0257 *	2.102	0.149	2.782	0.0972^†^
MGS success rate		4.413	0.0352 *	0.754	0.3863	0.041	0.8407
**C.** Correlation among saccade parameters in GTS patients.							
Latency	VGS	**-**					
	MGS	0.599 *	**-**				
Accuracy	VGS	−0.036	−0.121	-			
	MGS	−0.376 *	−0.595 *	0.35 *	-		
Frequency of saccades to the cue		0.154 *	0.436 *	−0.162 *	−0.426 *	-	
MGS success rate		−0.455 *	−0.689 *	0.15	0.655 *	−0.469 *	-
		Latency		Accuracy		Frequency of saccades to the cue	MGS success rate
		VGS	MGS	VGS	MGS		

*: *p* < 0.05 ^†^: *p* < 0.1.

## Data Availability

Data are contained within the article.
